# A prediction model for the efficacy of continuous positive airway pressure on bronchiolitis

**DOI:** 10.3389/fped.2022.1033992

**Published:** 2022-11-29

**Authors:** Qingxia Shi, Zhihua Zhao, Jilei Lin, Yin Zhang, Jihong Dai

**Affiliations:** ^1^ Department of Respiratory, Children’s Hospital of Chongqing Medical University, National Clinical Research Center for Child Health and Disorders, Ministry of Education Key Laboratory of Child Development and Disorders, Chongqing, China; ^2^ Children's Hospital of Chongqing Medical University, Chongqing Key Laboratory of Pediatrics, Chongqing, China; ^3^ Department of Respiratory Medicine, Shanghai Children’s Medical Center, Shanghai Jiaotong University School of Medicine, Shanghai, China; ^4^ Department of Respiratory and Critical Care Medicine, West China Hospital of Sichuan University, Sichuan, China

**Keywords:** continuous positive airway pressure, bronchiolitis, prediction model, children, treatment

## Abstract

**Objectives:**

Prediction of the efficacy of continuous positive airway pressure (CPAP) on bronchiolitis is necessary for timely treatment. This study aims to establish a nomogram for efficacy of CPAP on bronchiolitis, and compares accuracy with Pediatric Risk of Mortality III (PRISM III), Brighton Pediatric Early Warning Score (Brighton PEWS) and Pediatric Critical Illness Score (PCIS).

**Methods:**

From February 2014 to December 2020, data on children diagnosed with bronchiolitis and treated with CPAP in Chongqing was collected. The nomogram was evaluated by using multivariate logistic regression analysis. We compared the predictive value of model with PRISM III, PEWS and PCIS.

**Results:**

A total of 510 children were included. The nomogram prediction model including fever, APTT, white blood cells, serum potassium concentration, lactic acid, immunodeficiency, atelectasis, lung consolidation, congenital airway dysplasia and congenital heart disease was established. The AUC of the nomogram was 0.919 in the training set and 0.947 in the validating set. The model fitted well, as evidenced by the calibration curve and Hosmer-Lemeshow goodness-of-fit test. We discovered that the nomogram significantly performed better than PRISM III, PCIS and PEWS.

**Conclusions:**

A nomogram including ten factors for predicting the efficacy of CPAP on bronchiolitis was established. It had higher performance than the PRISM III, PCIS, and PEWS in terms of clinical benefits.

## Introduction

Bronchiolitis is a respiratory disease specific to children under 2 years old, which peaks in the fall and winter (1). It is a major cause of illness and hospitalization in infants and children. Hospital costs in the US exceed $734 million (2).

Studies have demonstrated that continuous positive airway pressure (CPAP) and high-flow nasal cannula (HFNC) can enhance physiologic and clinical outcomes associated with respiratory distress and failure due to bronchiolitis (3–5). However, mechanical ventilation is more appropriate for infants who have worsening severe distress despite non-invasive ventilation. Several studies suggest that CPAP improves ventilation and avoids endotracheal intubation (6, 7). Therefore, predicting the efficacy of CPAP on bronchiolitis will help improve physiologic and clinical outcomes. To our knowledge, bronchiolitis can cause symptoms such as fever, cough and wheezing. A domestic study found that P/F after 2 h of CPAP treatment, atelectasis and cardiac insufficiency can predict CPAP treatment failure in children with bronchiolitis (8). In general, Combining clinical manifestations, laboratory indicators and complications can be used to predict the efficacy of CPAP on bronchiolitis. Besides, the Pediatric Risk of Mortality III (PRISM III), Brighton Pediatric Early Warning Score (Brighton PEWS), and Pediatric Critical Illness Score (PCIS) are used to assess the condition of critically ill children. There is a lack of application of a scoring system in studies of CPAP efficacy prediction for bronchiolitis.

We aim to develop a predictive tool for CPAP treatment failure in children with bronchiolitis. We also compare the predictive value of model with PRISM III, PEWS and PCIS.

## Materials and methods

### Data source

According to the inclusion and exclusion criteria, a case-control study was conducted from February 2014 to December 2020 at the Respiratory Department, Children's Hospital of Chongqing Medical University. It was used to develop the prediction model. The validation data set comprised children with bronchiolitis between January 2021 and June 2022 in the Children's Hospital of Chongqing Medical University. All participants' guardians have signed the “Basic Informed Consent of Inpatients”. Our study complies with medical ethics standards.

### Study population

Inclusion criteria: (1) children who were clinically diagnosed with bronchiolitis younger than 2 years old, (2) Grading of severity: moderate to severe (9), (3) Nasal prongs CPAP therapy was supplied after admission.

Exclusion criteria: (1) mechanical ventilation immediately after admission, (2) ventilator care outside of the hospital, (3) the information was insufficient, (4) the guardian refused to continue the treatment.

Children who were weaned within 48 h after CPAP treatment were included in the success group, while children who needed to take endotracheal intubation within 48 h after CPAP treatment were included in the failure group. The positive continuous pressure of CPAP almost ranged from 2 to 6 cmH_2_0. The fraction of oxygen in the gas flowing in the system was subsequently adjusted to maintain a SpO_2_ of 95% or more. In the first 48 h after admission, we would give drugs such as budesonide and bronchodilators for symptomatic treatment. In addition, the drug treatment plan would be adjusted according to the examination results of patients.

### Data extraction

Two people independently collected the clinical symptoms, laboratory values and radiological data of hospitalized children. Within 24 h of admission, two individuals independently documented PRISM III, PCIS, and PEWS-related variables. If some indicators occurred more than once within 24 h, the most recent data before CPAP use was chosen. ALL data used for prediction were extracted before commencement of CPAP.

### Definitions

The PRISM III includes 17 physiological parameters such as heart rate, temperature, white blood cells (WBC) and so on (10). The higher the score, the more severe the disease. The PCIS includes 11 items including respiration, PaO_2_ and PH. The lower the score, the more severe the disease (11). The Brighton PEWS includes three aspects: consciousness, cardiovascular and respiration (12). The severity of the disease is correlated with the score. Malnutrition was defined as weight loss and subcutaneous fat loss. Liver damage was defined as the elevation of enzymology or bilirubin. Myocardial damage was diagnosed through clinical symptoms, signs and laboratory tests. Usually, children with myocardial damage will show shortness of breath, long sighs, and pale complexion. Laboratory tests showed that the serum and myocardial enzymes were elevated in the acute phase. Renal insufficiency was defined as the decline of renal function with or without oliguria or anuria.

### Statistical analysis

The sample size was determined using the PASS15.0 application. Normally distributed continuous data were compared by independent samples t-test and were expressed as mean ± standard deviation (M ± SD). Nonnormally distributed continuous data were compared using the Mann-Whitney U test and expressed as median (upper and lower quartiles) [P50(P25-P75)]. Categorical data were analyzed by the *χ*^2^ test or Fisher's exact test and were presented as numbers (*n*) and percentages (%). The factors influencing the efficacy of CPAP on children with bronchiolitis were investigated using logistic regression analysis. When *p*-value < 0.05, the difference was statistically significant. The prediction value of three scoring systems was assessed using the receiver operating characteristic (ROC) curve.

The picked predictors were presented with a nomogram in a training set and then validated in another data set. To verify the accuracy of nomogram, the area under the curve (AUC) of ROC was employed. The calibration curve and the Hosmer-Lemeshow test were used to assess the predictive performance of the nomogram. All data analyses were performed using SPSS statistics V.25.0 and R V.3.6.1 software.

## Results

### Clinical characteristics of included children

**Figure 1:**
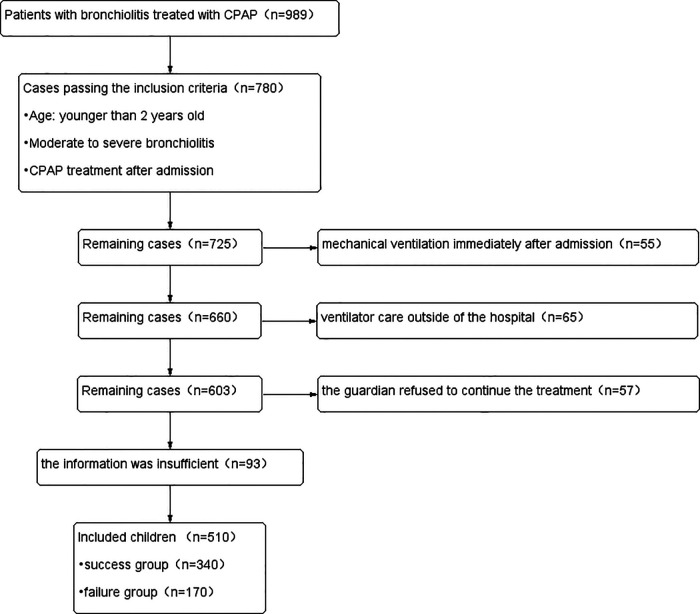
Study flow diagram.

510 children with bronchiolitis were eventually included in this study based on the inclusion and exclusion criteria, as well as the results of the sample size calculated by the PASS 15.0 software (Figure 1). The success group had 340 cases and the failure group had 170 cases in the training set. 250 children were divided into the validating set. The success group had 170 cases and the failure group had 80 cases in the validating set. Children in failure group were older than those in success group (*p* = 0.000). Birthweight, cough, fever, L, Plt, ALB, Na+, K+, lactic acid (Lac), PaO_2_, PH, PCIS were significantly lower, while procalcitonin, WBC, LDH, blood urea nitrogen, APTT, PaCO_2_, PRISM III, PEWS were significantly higher in the failure group than the success group. Premature, breastfeeding, myocardial damage were lower in failure group. Immunodeficiency, malnutrition, congenital airway dysplasia (CAD), bronchopulmonary dysplasia, congenital heart disease, atelectasis, pneumothorax, lung consolidation, pulmonary hypertension, liver damage, renal insufficiency were different in two groups. Demographic, clinical characteristics, laboratory findings and complications of the patients are summarized in Table 1.

**Table 1:** Clinical data of two groups of children with bronchiolitis.

	Total (*n* = 510)	Success (*n* = 340)	Failure (*n* = 170)	*p*
**Cardinal data**				
Sex [male/female, *n*]	365/145	248/92	117/53	0.331
Age [P50 (P25–P75), m]	3.50 (2.10–6.33)	3.00 (2.00–5.33)	4.63 (2.43–8.97)	0.000
Weight [P50 (P25-P75), kg]	6.00 (5.00–7.70)	6.00 (5.00–7.50)	6.00 (4.50–7.98)	0.089
Birthweight [P50 (P25-P75), kg]	3.05 (2.60–3.40)	3.10 (2.70–3.40)	3.00 (2.31–3.40)	0.014
Premature [*n* (%)]	115 (22.50%)	66 (19.40%)	49 (28.80%)	0.017
Cesarean section [*n* (%)]	280 (54.90%)	191 (56.20%)	89 (52.40%)	0.413
Breastfeeding [*n* (%)]	319 (62.50%)	196 (57.60%)	123 (72.40%)	0.001
**Clinical manifestation**				
Cough [P50 (P25-P75), day]	5 (3–10)	5 (3–7)	5.5 (3–13)	0.015
Gasp [P50 (P25-P75), day]	3 (1–4)	3 (2–4)	3 (1–5)	0.792
Fever [P50 (P25-P75), day]	0 (0–0)	0 (0–0)	0 (0–1)	0.005
**Basic illness [*n* (%)]**				
Immunodeficiency	13 (2.50%)	2 (0.60%)	11 (6.50%)	0.000
Malnutrition	31 (6.10%)	7 (2.10%)	24 (14.1%)	0.000
Congenital airway dysplasia	21 (4.10%)	4 (1.20%)	17 (10.00%)	0.000
Bronchopulmonary dysplasia	34 (6.70%)	6 (1.80%)	28 (16.50%)	0.000
Congenital heart disease	148 (29.00%)	73 (21.50%)	75 (44.10%)	0.000
21 trisomy syndrome	5 (1.00%)	2 (0.60%)	3 (1.80%)	0.427
**Laboratory indicator [P50 (P25-P75)]**				
Procalcitonin [*10^9^/L]	0.11 (0.07–0.22)	0.11 (0.07–0.20)	0.12 (0.07–0.53)	0.070
WBC count [ *10^9^/L]	9.29 (7.03–12.58)	9.01 (6.86–11.83)	9.75 (7.38–15.01)	0.002
Lymphocyte ratio [%]	0.51 (0.35–0.62)	0.54 (0.40–0.64)	0.43 (0.28–0.58)	0.000
Platelets [*10^9^/L]	425.00 (326.00–524.00)	432.00 (339.00–527.00)	403.50 (307.50–515.50)	0.030
Hemoglobin [g/L]	106.00 (97.00–115.00)	106.00 (98.00–114.00)	106.00 (95.00–116.00)	0.660
ALT [U/L]	34.70 (26.00–45.20)	35.00 (27.00–46.90)	33.45 (25.33–44.88)	0.129
AST [U/L]	42.00 (34.20–56.00)	41.90 (34.00–55.00)	42.70 (35.48–66.45)	0.068
Albumin [g/L]	40.60 (37.70–43.80)	40.80 (38.00–43.90)	40.05 (36.55–43.40)	0.026
LDH [U/L]	269.00 (223.60–324.00)	259.00 (216.80–309.00)	293.25 (233.40–368.50)	0.000
Creatinine [umol/L]	21.60 (18.30–25.00)	21.70 (18.30–24.60)	21.25 (18.35–25.93)	0.357
Blood urea nitrogen [mmol/L]	2.57 (1.88–3.54)	2.48 (1.70–3.30)	2.90 (2.10–4.38)	0.000
APTT [s]	28.00 (25.00–34.10)	26.30 (24.30–30.00)	34.10 (27.83–39.28)	0.000
Na + [mmol/L]	138.80 (136.60–140.70)	139.00 (136.80–140.90)	138.40 (136.23–140.10)	0.027
K + [M ± SD, mmol/L]	4.86 ± 0.66	4.94 ± 0.62	4.71 ± 0.70	0.000
Lactic acid [mmol/L]	2.10 (1.40–3.10)	2.30 (1.40–3.30)	2.00 (1.30–2.60)	0.002
PaCO_2_ [mmHg]	43.00 (36.00–50.00)	42.00 (35.00–49.00)	45.00 (38.00–53.00)	0.000
PaO_2_ [mmHg]	77.00 (62.00–96.00)	79.00 (66.00–104.00)	68.50 (55.25–85.75)	0.000
PH	7.37 (7.33–7.40)	7.38 (7.34–7.41)	7.36 (7.31–7.40)	0.001
TCO_2_ [mmol/L]	25.20 (22.70–28.60)	25.40 (22.50–28.50)	25.05 (23.00–29.65)	0.184
Glucose [mmol/L]	6.00 (5.30–7.00)	6.00 (5.30–6.80)	6.20 (5.20–7.10)	0.168
PRISM III [*n*]	0 (0–2)	0 (0–0)	3 (1–7.75)	0.000
PEWS [*n*]	3 (2–4)	2 (2–4)	4 (3–5)	0.000
PCIS [*n*]	90 (86–92)	92 (88–96)	88 (84–90)	0.000
**Complication [*n* (%)]**				
Pulmonary hemorrhage	1 (0.20%)	0 (0.00%)	1 (0.60%)	0.333
Atelectasis	77 (15.10%)	14 (4.10%)	63 (37.10%)	0.000
Pneumothorax	7 (1.40%)	1 (0.30%)	6 (3.50%)	0.011
Lung consolidation	89 (17.50%)	11 (3.20%)	78 (45.90%)	0.000
Pulmonary hypertension	43 (8.40%)	14 (4.10%)	29 (17.10%)	0.000
Liver damage	88 (17.30%)	43 (12.60%)	45 (26.50%)	0.000
Myocardial damage	183 (35.90%)	107 (31.50%)	76 (44.70%)	0.003
Renal insufficiency	5 (1.00%)	0 (0.00%)	5 (2.90%)	0.007

WBC, white blood cell; ALT, alanine aminotransferase; AST, aspartate aminotransferase; LDH, lactate dehydrogenase; APTT, Activated partial thromboplastin time; Na+, sodium; K+, potassium; PaCO_2_, pressure of carbon dioxide; PaO_2_, partial pressure of oxygen; TCO_2_, total carbon dioxide; PRISM III, pediatric risk of mortality III; PEWS, pediatric early warning score; PCIS, pediatric critical illness score.

In univariate and multivariate logistic regression analysis, fever, APTT, WBC, serum potassium concentration, Lac, immunodeficiency, atelectasis, lung consolidation, CAD, congenital heart disease (CHD), PRISM III, PCIS, and PEWS were the influencing factors of the efficacy of CPAP on bronchiolitis (Tables 2, 3).

**Table 2:** Univariate logistic regression analysis of the efficacy evaluation of bronchiolitis.

	*p*	OR	95%CI
Age	0.000	1.154	1.093–1.220
Birthweight	0.002	0.656	0.503–0.856
Premature	0.017	0.595	0.388–0.912
Breastfeeding	0.001	0.520	0.349–0.775
Cough	0.000	1.078	1.042–1.115
Fever	0.016	1.129	1.023–1.247
Immunodeficiency	0.002	0.086	0.019–0.390
Malnutrition	0.000	0.128	0.054–0.303
Congenital airway dysplasia	0.000	0.107	0.035–0.324
Bronchopulmonary dysplasia	0.000	0.091	0.037–0.225
Congenital heart disease	0.000	0.346	0.233–0.516
WBC	0.000	1.072	1.037–1.109
Lymphocyte ratio	0.000	0.034	0.011–0.106
Leukocyte	0.079	0.999	0.998–1.000
Platelets	0.002	0.943	0.910–0.978
LDH	0.000	1.005	1.003–1.007
Blood urea nitrogen	0.000	1.280	1.139–1.439
APTT	0.000	1.169	1.130–1.210
Na+	0.110	0.963	0.919–1.009
K+	0.000	0.572	0.428–0.766
Lactic acid	0.003	0.783	0.667–0.919
PaCO_2_	0.000	1.041	1.022–1.059
PaO_2_	0.000	0.985	0.978–0.992
PH	0.000	0.003	0.000–0.075
PRISM III	0.000	1.648	1.478–1.838
PEWS	0.000	2.370	1.975–2.846
PCIS	0.000	0.799	0.760–0.839
Atelectasis	0.000	0.073	0.039–0.135
Pneumothorax	0.020	0.081	0.010–0.675
Lung consolidation	0.000	0.039	0.020–0.077
Pulmonary hypertension	0.000	0.209	0.107–0.407
Liver damage	0.000	0.402	0.252–0.642
Myocardial damage	0.003	0.568	0.389–0.830
Renal insufficiency	0.999	0.000	0.000–0.000

CI, confidence interval; WBC, white blood cell; LDH, Lactate dehydrogenase; APTT, Activated partial thromboplastin time; Na+, Sodium; K+, Potassium; PaCO_2_, Pressure of carbon dioxide; PaO_2_, Partial pressure of oxygen; PRISM III, Pediatric Risk of Mortality III; PEWS, Pediatric Early Warning Score; PCIS, Pediatric Critical Illness Score.

**Table 3:** Multivariate logistic regression analysis of the efficacy evaluation of bronchiolitis.

	*p*	OR	95%CI
Fever	0.018	1.392	1.058–1.831
APTT	0.000	1.282	1.177–1.397
WBC	0.001	1.190	1.078–1.314
K+	0.033	0.444	0.210–0.938
Lactic acid	0.010	0.589	0.394–0.880
Atelectasis	0.023	0.237	0.068–0.822
Lung consolidation	0.012	0.207	0.061–0.706
Congenital airway dysplasia	0.004	0.077	0.013–0.445
Congenital heart disease	0.041	0.353	0.130–0.958
Immunodeficiency	0.031	30.964	1.378–695.976
PRISM III	0.000	1.524	1.295–1.793
PCIS	0.002	0.878	0.810–0.951
PEWS	0.000	1.788	1.292–2.476

CI, confidence interval; APTT, activated partial thromboplastin time; WBC, white blood cell; K+, potassium; PRISM III, pediatric risk of mortality III; PEWS, pediatric early warning score; PCIS, pediatric critical illness score.

### Developing a nomogram in the training set

**Figure 2:**
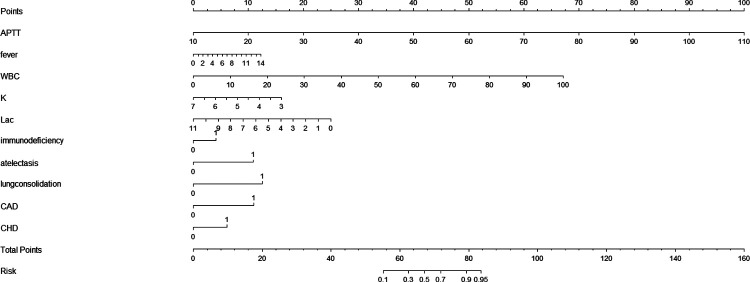
Nomogram for prediction of CPAP treatment for bronchiolitis.

**Figure 3:**
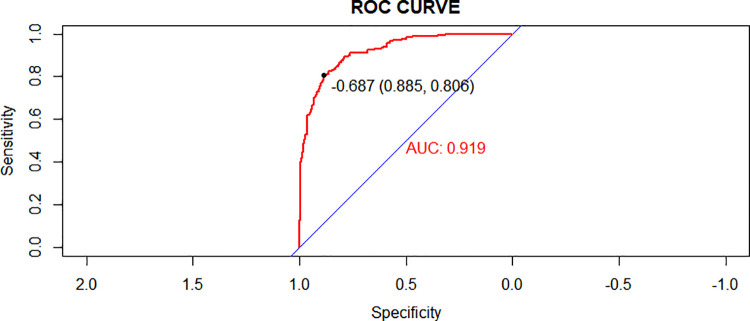
Receiver operating characteristic (ROC) curves of the nomogram model in the training cohort.

**Figure 4:**
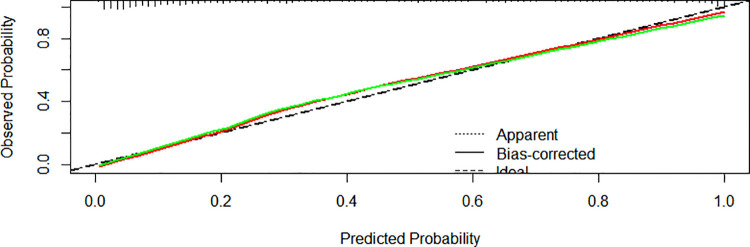
Calibration curves of the nomogram model in the training cohort.

We created a nomogram that included statistically significant influencing factors (Figure 2). The nomogram revealed that immunodeficiency, atelectasis, CAD, lung consolidation and CHD had a risk score of 4.5, 12, 12, 13, 6. The duration of fever, APTT and WBC risk score all increased with the actual value of treatment failure in children with bronchiolitis. In contrast, the risk scores for potassium and lactate increased with decreasing actual value of treatment failure in patients. In the training set's nomogram, the AUC for the probability of CPAP treatment failure in children with bronchiolitis was 0.919 (0.895–0.943) (Figure 3). The calibration curve for the model revealed high agreement between prediction and observation in the training cohort (Figure 4). The *p*-value of Hosmer-Lemeshow test was 0.2707, which indicated that the model fitted well.

### Validation of the nomogram

**Figure 5:**
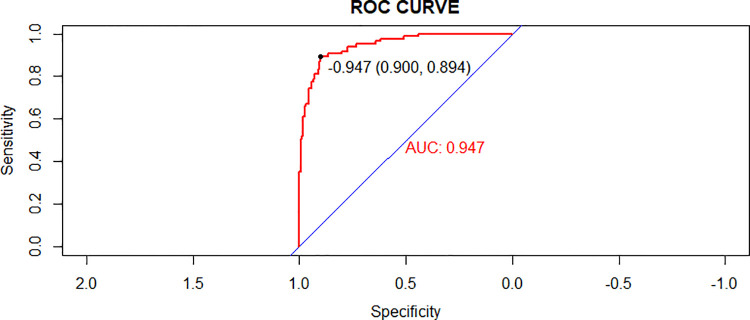
Receiver operating characteristic (ROC) curves of the nomogram model in the validation cohort.

**Figure 6:**
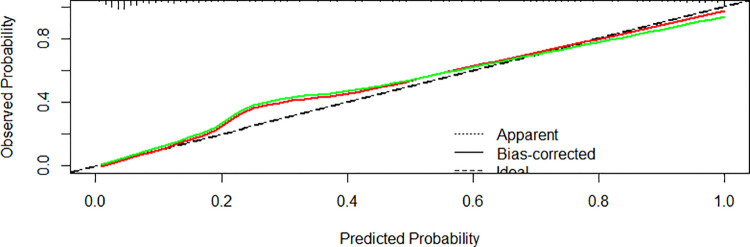
Calibration curves of the nomogram model in the validation cohort.

In the validating set, the AUC for the probability of CPAP treatment failure for bronchiolitis was 0.947 (0.921–0.974) (Figure 5). Hosmer-Lemeshow goodness-of-fit tests revealed no significant difference between observed and predicted events (*p *= 0.8606) (Figure 6).

### Comparison with three critical scores

**Figure 7:**
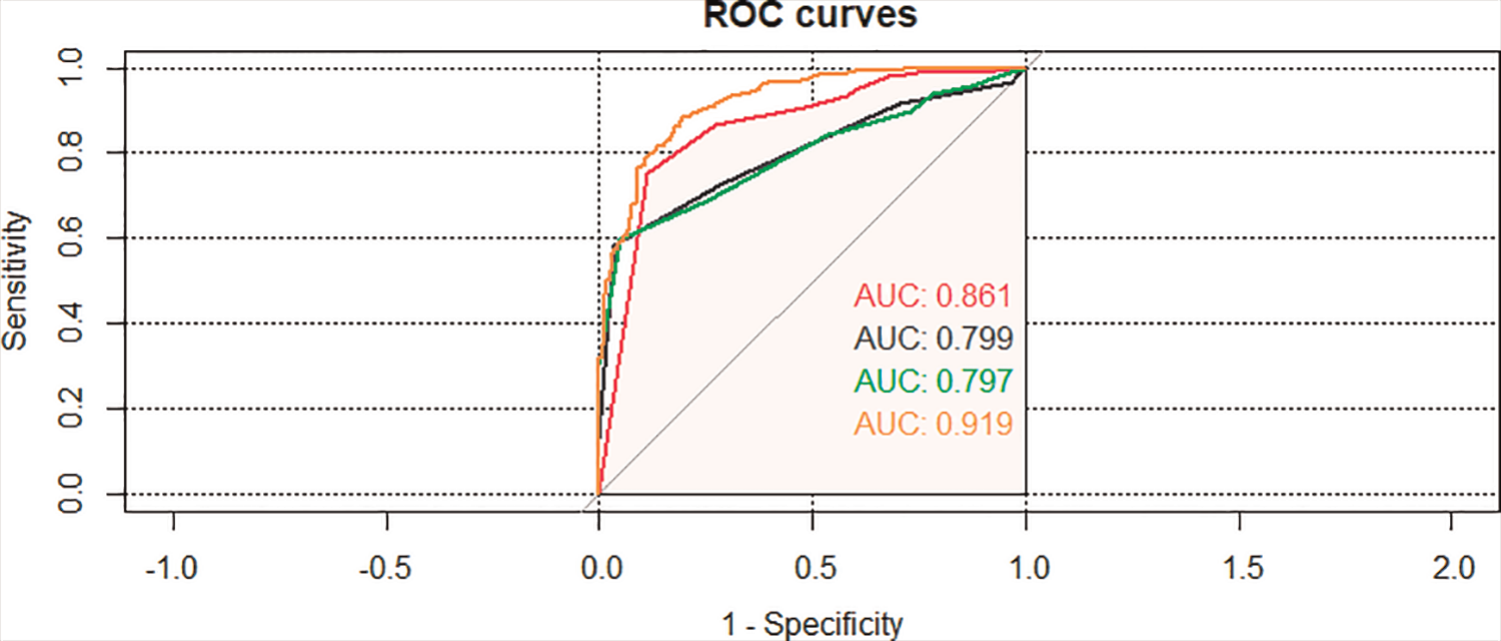
Area under the curve (AUC) of comparison of the nomogram and the three critical scores in the validating set.

For all included children, PRISM III, PCIS, and PEWS had an AUC of the probability of CPAP treatment failure on bronchiolitis of 0.861 (0.825–0.896), 0.797 (0.760–0.835), 0.799 (0.762–0.837) (Figure 7, Table 4). The results indicated that three scores can predict the efficacy of CPAP on bronchiolitis to a certain extent. To clarify the clinical utility of nomogram, we compared the AUC of nomogram with the curves of three scoring systems. The predictive power was comparable to PEWS and PCIS (*p *= 0.937), but none of them was as accurate as PRISM III (*p *< 0.05). The AUC of nomogram was significantly bigger than PRISM III, PCIS, and PEWS (all *p *< 0.05).

**Table 4:** ROC analysis of three scoring systems for treatment failure of bronchiolitis.

Scoring system	AUC	Youden index	Sensitivity	Specificity	95%CI	*p*
PRISM III	0.861	0.641	88.8%	75.3%	0.825–0.896	0.000
PCIS	0.797	0.550	59.7%	95.3%	0.760–0.835	0.000
PEWS	0.799	0.550	96.5%	58.5%	0.762–0.837	0.000

AUC, area under the curve; CI, confidence interval; PRISM III, pediatric risk of mortality III; PEWS, pediatric early warning score; PCIS, pediatric critical illness score.

## Discussion

We developed and validated a predictive model to predict CPAP treatment for bronchiolitis. The prediction model incorporates fever, APTT, WBC, serum potassium concentration, Lac, immunodeficiency, atelectasis, lung consolidation, CAD and CHD. It shows good accuracy, which indicates that nomogram may has good utility in clinical practice. This nomogram was more accurate than PRISM III, PEWS and PCIS.

CPAP is a valuable non-invasive ventilation technique that can reduce work of breathing and improve gas exchange (13). Jat et al.'s study discovered CPAP can reduce respiratory rate when compared to standard treatment (3). Furthermore, CPAP has been shown in certain trials to significantly improve respiratory symptoms (6, 14). In general, it's important to investigate factors that contribute to CPAP treatment failure on bronchiolitis.

In this study, fever, APTT, WBC, serum potassium concentration, Lac, immunodeficiency, atelectasis, lung consolidation, CAD, CHD, PRISM III, PEWS, PCIS were the influencing factors of CPAP treatment failure on bronchiolitis. The findings may have implications for clinical practice. Fever and WBC count both represent the inflammatory reaction of the body. The clinical manifestations of bronchiolitis include cough, wheezing and fever (1). Prolonged fever indicates that patients are suffering from severe disease with a long course. The WBC count will increase when there is infection and inflammatory reaction is intense, which is a defensive reaction of the body. Studies have shown that WBC counts can be used to predict and diagnose the severity of bronchiolitis (15). The rise of APTT indicates that the blood is in a hypercoagulable state, which is caused by the release of various inflammatory factors and clotting substances during infection (16). The hypercoagulable state of blood will cause pulmonary microvascular contraction and blood block, which leads to aggravation of pulmonary and respiratory diseases. The study (17) showed that hypokalemia was a common electrolyte abnormality in pneumonia. Hypokalemia can cause damage to the body, such as abdominal distension, arrhythmia, dyspnea, etc. Changes in Lac can lead to imbalance in patient's internal environment. Bronchiolitis will cause changes in serum metabolism of patients. The study discovered that the risk factors for prolonged hospitalization of children with bronchiolitis were cardiac abnormalities, airway abnormalities and immune abnormalities (18, 19). Atelectasis and lung consolidation are well-recognized factors for severity of disease with bronchiolitis. They are clinically established indicators of disease severity. When patients are complicated by CAD or CHD, immunodeficiency, atelectasis, or lung consolidation, airway function will be impaired. Therefore, close monitoring risk factors, comprehensive treatment measures and oxygen supply after maintaining airway patency are the key to treatment success.

PRISM III is the most widely used pediatric critical care score. Kaur's study proved that it is capable of accurately determining the severity of the disease and predicting prognosis (20). Mayordomo-Colunga et al.'s study found that higher PRISM III was associated with non-invasive ventilation treatment failure (21). Our findings were similar to theirs. Furthermore, the result revealed that PRISM III had high sensitivity and specificity. This could be because PRISM III includes important physiological parameters from multiple systems. However, it is impractical to dynamically monitor all physiological parameters in clinical practice.

PEWS is simple and convenient, and it can serve as a useful tool for clinicians to evaluate the condition of children effectively. Therefore, it was suggested in the UK's 2015 guidelines on bronchiolitis (22). Our study also confirmed that PEWS is useful to predict the efficiency of CPAP on bronchiolitis. But the specificity of PEWS was low, which may be the limited scoring content.

PCIS is a pediatric critical care score that is most frequently used in China. Secondary hospitals can acquire pertinent data for scoring. A study suggested that PCIS might be utilized to evaluate the severity of disease and forecast prognosis, which is similar to our study (23). In addition, the results demonstrated that PCIS's predictive value is inferior to PRISM's, which was consistent with the findings of earlier investigations (24). This suggests that PCIS has low sensitivity in predicting CPAP treatment failure on bronchiolitis.

In our study, the performance of the prediction model established is significantly better than three scoring systems. We have to admit there is a fundamental bias in our research. We are comparing 3 all-comers (including any disease or disorder) scoring systems in a disease-specific study population with a disease-specific nomogram that we have formulated from the disease-specific data of that specific study group. We take into account factors including pulmonary problems and the length of the illness to build a nomogram. The nomogram model can assist us in intuitively comprehending the impact of numerous influencing factors at various levels on the effectiveness of CPAP on bronchiolitis. Furthermore, data of the predictive model are available for clinical use and can be applied to hospitals at different levels.

This study had several strengths. It's the first time to build a prediction model for CPAP treatment failure on bronchiolitis. There is a lack of tools about CPAP treatment on bronchiolitis. In practical clinic physicians need a prediction model for CPAP treatment failure on bronchiolitis. Three scoring systems for assessing the effectiveness of CPAP on bronchiolitis has also never been studied before. It is encouraging that three scoring systems can also predict the efficacy of CPAP on bronchiolitis. The nomogram performed better in predicting efficiency. This study is useful for clinical work because it identifies influencing factors that contribute to CPAP treatment failure on bronchiolitis. Although the scoring method might be challenging to implement in actual work settings, the nomogram is rather straightforward and might gain popularity over time. The limitation of this study is that it is a single-center case-control study, which may lead to heterogeneity of results. The physiological characteristics and outcomes may change as bronchiolitis in children progresses under various situations. The numerical variation of nomogram may be statistically relevant, but in real clinical practice would not be so different. Therefore, more research is needed in the future to find more indicators applicable to clinical practice.

## Conclusion

In this study, we found that fever, APTT, WBC, serum potassium concentration, Lac, immunodeficiency, atelectasis, lung consolidation, CAD, CHD, PRISM III, PCIS, and PEWS were the influencing factors of the efficacy of CPAP on bronchiolitis. Compared with the three scoring systems, nomogram composed of the remaining independent risk factors had higher predictive power.

## Data Availability

The original contributions presented in the study are included in the article/Supplementary Material, further inquiries can be directed to the corresponding author/s.
